# Suivi prospectif sur 5 ans des tentatives de suicide en population clinique dans la région de Fès, Maroc

**DOI:** 10.11604/pamj.2014.18.321.3726

**Published:** 2014-08-21

**Authors:** Chadya Aarab, Fatima Elghazouani, Rachid Aalouane, Ismail Rammouz

**Affiliations:** 1 Centre psychiatrique universitaire IbnAlhassan, CHU Hassan II, Fès, Maroc

**Keywords:** Tentative de suicide, facteurs de risque, récidive suicidaire, suivi longitudinal, Suicide attempt, risk factors, recurrent suicidal, longitudinal follow-up

## Abstract

Au Maroc, les tentatives de suicide (TS) demeurent un sujet mal évalué à cause de considérations socioculturelles et l'absence d'approche longitudinale. L'objectif principal était d’évaluer le devenir des suicidants après 5ans au centre universitaire psychiatrique de Fès, les objectifs secondaires étaient l'estimation de la prévalence des TS, préciser les étiologies les plus fréquentes, et établir une corrélation entre les paramètres sociodémographiques, cliniques et évolutifs. Etude prospective à visée transversale et longitudinale, incluant les suicidants vus à l'hôpital psychiatrique de Fès, avec un suivi longitudinal sur 5ans. L’évaluation a été faite par un hétéro-questionnaire et le Mini International Neuropsychiatric Interview (MINI) cherchant le trouble psychiatrique sous jacent. On a recruté 105 patients suicidants, 62% des femmes, l’âge moyen est de 29,23ans. La prévalence des tentatives de suicide est de 3% sur l'ensemble des consultants à l’établissement. Les troubles de l'humeur, psychotiques et de personnalité ont occupé respectivement 37,6%, 27,7%, et 25,7% des cas. La récidive suicidaire a été notée chez 54% des patients, était significativement corrélée à la vie solitaire (P: 0,039) et à la présence d'antécédents familiaux de TS (P: 0,046). L'utilisation de moyens violents était significativement fréquente chez les patients psychotiques. Après 5ans, 32 patients ont répondu à notre appel. Le taux de récidive était de 15%. On a noté trois cas de décès dont deux suicides confirmés. Les résultats confirment les données de la littérature scientifique avec certaines particularités cliniques et évolutives.

## Introduction

Les conduites suicidaires représentent un problème sérieux de santé publique dans la majorité des pays. Elles suscitent de plus en plus d'intérêt de tous les professionnels de la santé. Ce phénomène complexe prend son importance vu l'augmentation des accidents de suicide et des tentatives de suicide avec leurs conséquences médicales et psychosociales à cours et à long terme. Plusieurs études ont proposé différentes méthodes d'intervention pour la prévention du suicide à différents niveaux. [1, 2]. Au Maroc, le thème de suicidalité est difficile à aborder à cause des facteurs religieux et socioculturels. On ne dispose pas de chiffres de prévalence des conduites suicidaires à l’échelle nationale, toutefois des études transversales au niveau des centres hospitaliers psychiatriques ont avancé des résultats concernant des populations cliniques. La seule étude réalisée en population générale, est celle d'Agoub et coll ayant objectivé une prévalence des tentatives de suicide de 2,1% et des idéations suicidaires de 6,3% [3].

Les études de suivi longitudinal des tentatives de suicide sont peu nombreuses en littérature et inexistantes au Maroc. Elles portent un intérêt particulier pour l’évaluation des facteurs de risque prédictifs de récidive. Ainsi notre travail vise à étudier la prévalence des tentatives de suicide, déterminer les principales affections psychiatriques associées, établir une corrélation entre les différents paramètres sociodémographiques, cliniques et évolutifs avec le risque suicidaire, et enfin un objectif capital qui consiste à évaluer le taux de la récidive suicidaire sur 5ans au sein de la population étudiée, ainsi que l'insertion socioprofessionnelle et le niveau d'observance médicamenteuse relative au trouble psychiatrique sous jacent.

## Méthodes

C'est une étude prospective qui vise à dégager une approche transversale et longitudinale des tentatives de suicide (TS) dans la région de Fès et qui dispose d'une population de 3 millions d'habitants. L’étude a eu lieu au niveau du centre psychiatrique universitaire « Ibn Alhassan ». La durée de recrutement des malades a été étalée sur 32 mois entre avril 2006 et décembre 2008. La Population cible a inclut toute personne venant pour consultation ou hospitalisation aux urgences du centre psychiatrique pour un motif de tentative de suicide quelque soit la nature de la maladie et quelque soit l’âge. L’évaluation a été faite par des psychiatres aux urgences à l'aide d'un hétéro-questionnaire comportant au total 55 items. Il s'organise autour de trois thèmes: Une première partie concerne les caractéristiques socio-démographiques du suicidant, une deuxième comporte les facteurs cliniques tels que les antécédents de tentative de suicide et les antécédents de consultation ou hospitalisation psychiatriques personnels et familiaux, ainsi que la notion d'usage de substances&dot,&dot,&dot;et la dernière partie est réservée au geste suicidaire, ses moyens, son déroulement, ses circonstances, les motifs invoqués et les soins. Une autre partie du questionnaire issu du Mini International Neuropsychiatric Interview (MINI) cherchant le trouble psychiatrique sous jacent.

Après 5 ans du premier recrutement, on a essayé de contacter les malades par téléphone avec quatre questions bien précises concernant: la récidive suicidaire, l'insertion socioprofessionnelle, l'observance thérapeutique par rapport à la prise en charge de la maladie psychiatrique sous jacente et les événements stressants. La première tentative de suicide est qualifiée de TS index.

Les données ont été saisies sur le logiciel «Excel» et analysées sur le logiciel «SPSS» version 17. Une analyse descriptive a été faite. Les variables qualitatives ont été présentées sous forme de pourcentage et les variables qualitatives sous forme de moyennes ± Ecart type. Pendant l'analyse univariée, la mesure d'association entre deux variables qualitatives a été réalisée par comparaison de pourcentages à l'aide du test de Chi-deux. La mesure d'association entre une variable quantitative et une autre qualitative a été réalisée par comparaison de deux ou plusieurs moyennes en utilisant le test de «Student» ou le test «Anova». Le risque ‘ de première espèce a été fixé à 5%.

**Aspect éthique**: le consentement verbal des malades a été accordé, la confidentialité des informations a été respectée en utilisant un questionnaire anonyme.

## Résultats

On a recruté 105 patients suicidants, 65 femmes (62%) et 40 hommes (38%), l’âge moyen est de 29,23ans, avec des âges extrêmes de 11ans et 60ans. La prévalence des tentatives de suicide est de 3% sur l'ensemble des consultants à notre établissement durant cette période. Les principales données sociodémographiques figurent dans le Tableau 1. Concernant les résultats cliniques, on a noté des antécédents psychiatriques riches chez la population d’étude, ainsi les troubles de l'humeur occupent le premier rang des pathologies psychiatriques. Le Tableau 2 montre les principales caractéristiques cliniques des suicidants.

**Tableau 1:** Données sociodémographiques des suicidants

Variables	Effectif	Pourcentage
**Genre**		
masculin	40	38%
féminin	65	62%
**Statut marital**		
célibataire	63	60%
Marié	27	26%
Divorcé, séparé, veuf	15	14%
Vit seul	17	16,2%
**Niveau d'instruction**		
Analphabète	32	32%
Primaire	34	34%
Secondaire,	24	24%
Universitaire	15	15%
Intégration Professionnelle absente	62	59%
**Niveau socio économique**		
Bas	62	59,8%
Moyen	33	31,4%
Haut	10	8,8%
**Lieu de vie**		
Urbain	83	79%
Rural	22	21%

**Tableau 2:** Données cliniques des suicidants

Variables	Pourcentage
ATCD personnels de TS	53,8%
ATCD personnels de consultation psychiatrique	49%
ATCD personnels d'hospitalisation	32%
ATCD personnels d'usage de substances	30,5%
ATCD familiaux de TS	13,7%
ATCD familiaux de suicide	7%
**Diagnostics psychiatriques**	
Troubles de l'humeur	37,6%
Troubles psychotiques	27,7%
Troubles de la personnalité	25,7%
Troubles anxieux	10,9%
Troubles addictifs	5%
Idéations suicidaires présentes	63%

Par rapport aux circonstances du geste suicidaire, l'acte suicidaire est survenu généralement dans le cadre de l'angoisse dans 48% des cas, de façon impulsive chez 41,2% des participants, de manière préméditée dans 7,8% des cas et enfin un contexte confusionnel a été noté dans 2,9% des situations. Concernant les moyens de tentative de suicide dans notre échantillon, l'intoxication médicamenteuse occupe le premier rang avec 25% des cas (dans les deux tiers, il s'agit de psychotropes); la défenestration vient en second choix chez nos patients (22%). A noter que les moyens violents (pendaison et défenestration) sont représentés dans un tiers des cas (33%).

De point de vue analytique, on a cherché certaines corrélations entre les différents paramètres. Le premier paramètre est la récidive suicidaire qui a été significativement corrélée à la vie solitaire (P: 0,039) et à la présence d'antécédents familiaux de tentative de suicide (P: 0,046). On n'a pas trouvé des résultats significatifs concernant les troubles psychiatriques, néanmoins le taux le plus élevé de récidive a été enregistré pour les troubles de l'humeur (43%). L'utilisation de moyens violents (la défenestration et pendaison) est fréquente et de façon significative chez les personnes ayant un trouble psychotique (56%), alors que ce chiffre est plus bas chez les suicidants ayant un troubles de la personnalité. Cette corrélation n'est pas significative pour les autres entités diagnostiques (Tableau 3). Les femmes utilisent largement des moyens non violents par rapport aux hommes. Mais ce résultat trouvé dans notre série n'est pas significatif. Par rapport au regret du geste suicidaire, les suicidants ayant des troubles de la personnalité regrettent plus et de façon significative le geste suicidaire par rapport aux autres troubles psychiatriques (p = 0.02).

**Tableau 3:** Usage de moyens violents selon les affections psychiatriques

Trouble psychiatrique	Moyens violents	P (indice de significativité)
Trouble psychotiques	56%	S (p =0.04)
Trouble humeur	30.3%	NS
Trouble addictifs	33%	NS
Troubles anxieux	20%	NS
Trouble de la personnalité	10%	S (p =0.01)

Après 5 ans, on a essayé de contacter les patients inclus dans l’étude, seulement 32 patients ont répondu à notre appel, ce qui représente 30,4% de l’échantillon initial. Pour les personnes injoignables ont dû changer leur numéro de téléphone. Au cours des cinq dernières années, 5 patients sur 32 ont refait une tentative de suicide (14%), le délai par rapport à la TS index est entre un an et quatre ans. Tous les suicidants ont utilisé les mêmes moyens qu'avant. On a noté trois décès dont deux suicides confirmés, le premier cas par intoxication médicamenteuse de psychotropes chez une femme suivie pour trouble dépressif récurrent en comorbidité avec une personnalité histrionique, le décès est survenu après deux ans de la TS index; le deuxième cas de suicide par défenestration chez un homme suivi pour une schizophrénie paranoïde survenu après un an de la TS index. Le troisième patient retrouvé mort en errance selon la famille. Les autres paramètres cherchés, l'insertion socioprofessionnelle est jugée suffisante chez 31% des participants et médiocre dans 25% des cas, l'observance médicamenteuse était bonne dans 56% des cas alors que la présence d’événements stressants est rapportée chez 22% des patients.

## Discussion

Le comportement suicidaire est un phénomène complexe qui implique des facteurs biologiques, psychologiques et sociaux. L'identification de facteurs de risque reste la principale stratégie pour la mise en ouvre de démarches de prévention.

Dans notre étude la prévalence des tentatives de suicide était de 3%, ce chiffre rejoint les données des études tunisiennes [4, 5], mais aussi ce qui est décrit dans la littérature occidentale où les tentatives de suicide représentent 2 à 4% des consultants dans les services des urgences [6]. Les résultats sociodémographiques dans notre étude sont en concordance avec les données de la littérature. L’âge moyen des suicidants dans notre travail est de 29,23 +/- 10 ans. Les tentatives de suicide sont l'apanage du sujet jeune et les adolescents en particulier [7]. Concernant le genre, il y a un accord à l'unanimité sur la prédominance féminine chez les suicidants, comme le montre une étude multicentrique sur 3206 suicidants qui a trouvé 67% de sujet féminin, une valeur proche de notre étude qui est de 62% [7]. Quant au statut marital, les célibataires constituent la catégorie la plus touchée comme le montre d'autres études [7]. On signale aussi une surreprésentation des patients inactifs dans notre série (59% des suicidants) ce qui est le cas d'autres études [8, 9]. Concernant les caractéristiques cliniques, 54% des suicidants ont déjà réalisé au moins une tentative de suicide, ce chiffre est comparable aux données de la littérature, rapportant des taux allant de 41% à 56% [10]. En effet, un antécédent de tentative de suicide constitue un facteur de risque pour une future conduite suicidaire et augmente aussi le risque de suicide fatal. Le risque est très augmenté dans l'année suivant l'acte mais reste plus élevé même à distance puisque 10% des sujets qui ont effectué une tentative de suicide décèdent par suicide dans les 10 ans. Les principaux facteurs de risque objectivés dans notre travail de récidive sont la vie solitaire et la présence d'antécédents familiaux de conduites suicidaires. Ces données rejoignent celles décrites dans d'autres études [11].

Les troubles psychiatriques demeurent le dénominateur commun le plus fortement associé aux conduites suicidaires, et bien que la constatation de cette association ne soit pas récente, elle a bénéficie d'un regain d'intérêt ces dernières années de la part des chercheurs. La plupart des études s'accordent sur le fait que plus de 90% des sujets ayant des conduites suicidaires souffraient d'un trouble psychiatrique [12]. Dans notre étude, la totalité des malades ont une affection psychiatrique caractérisée (axe I, II du DSM IV), et contrairement à ce que est retrouvé dans la majorité des études-où les troubles dépressifs occupent le premier rang, les troubles psychotiques, les troubles dépressifs et les troubles de la personnalité sont presque identiques représentant un peu près un tiers des cas pour chacun.

Selon des études prospectives, le risque de tentative de suicide au cours du trouble dépressif majeur est de 16% à 40% [13]. Les principaux facteurs prédictifs de suicide sont les antécédents personnels et/ou familiaux de conduite suicidaire, sujet jeune et le sexe féminin, dépression sévère récurrente, présence de symptômes psychotiques, désespoir, idéation suicidaire [14]. Les patients souffrant de troubles psychotiques constituent 27,7% de notre échantillon, ces derniers sont représentés essentiellement par la schizophrénie. Ce qui présente un chiffre élevé par rapport aux données de la littérature [5, 9]. Selon certaines études, 20 à 40% de ces patients font au moins une tentative de suicide au cours de leur vie, 10 à 15% des tentatives de suicide aboutissent contre 2% dans la population générale [15]. Les principaux facteurs de risque: sujet jeune, sexe masculin, dépression post psychotique, usage de substance et antécédents de conduite suicidaire [16].

Les troubles de personnalité occupent 25,7% des diagnostics dans notre travail, dont la plupart de nature histrionique. On note que la personnalité borderline dans notre série est faiblement présentée. Ce type de personnalité est fréquemment rapporté dans la littérature.

Le suicide est une continuité de l'idéation suicidaire, le plan suicidaire et la tentative de suicide. Dans notre étude, 63% de la population clinique avaient des idéations suicidaires avant le passage à l'acte. Shulberg et al rapportent que 19 à 54% des suicidants auraient évoqué des idées suicidaires avant le passage à l'acte [17]. Une enquête marocaine a évalué les conduites suicidaires dans un échantillon représentatif de la population générale, elle a objectivé la présence d'idéations suicidaires chez 6,3% des sujets, alors que 2,1% des participants ont signalé au moins une tentative de suicide au cours de sa vie [3].

Concernant la modalité de la tentative de suicide dans notre série, l'intoxication médicamenteuse en particulier par les psychotropes est présente dans 25% des cas. Le recours majoritaire à l'ingestion médicamenteuse a été retrouvé dans de nombreux travaux et ce pour des populations de cultures différentes [18]. Par ailleurs la défenestration est classée en 2ème position dans notre échantillon contrairement aux résultats de la littérature. Il n'y a pas de parallélisme strict entre la gravité de l'acte suicidaire, l'intensité du désir de mort et la gravité des perturbations psychopathologiques observées chez l'individu suicidant. Toutefois, l'importance de l'attaque corporelle (défenestration, etc) représente un facteur de gravité certain. Plusieurs études ont montré que parmi les meilleurs façons d'agir sur la prévention du suicide c'est la prévention secondaire basée sur le traitement et le suivi des personnes à risque en particulier qui présentent une pathologie psychiatrique et/ou ayant un antécédent de tentative de suicide [19]. Malgré les publications multiples concernant les conduites suicidaires, peu d’étude se sont intéressées au suivi longitudinal des suicidants. Cette rareté de publications est liée aux difficultés de suivi à long terme vu que les malades sont souvent perdus de vue ou refusent tout contact avec les psychiatres considéré stigmatisant. Ainsi la majorité des études sur des populations cliniques de suicidants s'oriente en général sur les facteurs de risque impliqués dans la récidive suicidaire. Dans notre série, le taux de récidive sur 5ans était de 15%, et trois cas de décès ont été notés. Ces résultats sont proches de ceux trouvés dans un travail réalisé sur un échantillon d'adolescents de 16 à 21 ans, avec évaluation en deux temps à six mois et à 18mois, les auteurs ont objectivé deux cas de décès violents et 18% des participants ont refait une tentative de suicide durant cette période [20]. Une autre étude multicentrique prospective, incluant les tentatives de suicide par intoxication médicamenteuse avec un suivi longitudinal sur 12mois a montré un taux de récidive de 30%. Les facteurs prédictifs retrouvés sont l’âge de 30 à 49ans, l'usage de substances en particulier les opiacés, le chômage, les tentatives de suicide antérieures et l'histoire de traitement psychiatrique [21]. Une étude prospective de suivi sur deux ans, incluant 273 suicidants a permis d'identifier les principaux facteurs de risque de récidive en particulier les antécédents de conduite suicidaire, l'usage de substance, l’état de stress post traumatique et l'arrêt de traitement [22]. Dans une revue de littérature Owens et coll ont montré que la prévalence des actes suicidaires variait de 15% -16% à 12mois avec augmentation à 20-25% au cours des années suivantes [23]. Une étude au Taiwan, a montré que le risqué de recidive suicidaire était de 5,7% au cours de la première année, et atteint 9,5% au bout de 4ans [24].

La plupart des études de suivi des suicidants se sont focalisées sur la répétition de l'acte suicidaire,alors que notre étude a eu l'avantage d’évaluer prospectivement en plus l'insertion socioprofessionnelle, la survenue d’événements stressants et l'observance thérapeutique ce qui fait l'originalité de notre travail. Dans notre série, l'insertion socioprofessionnelle est jugée globalement satisfaisante par les patients et leurs familles. Ces résultats ont été retrouvés par l’équipe de Légier qui a évalué sur dix ans le devenir psychosocial de 29 adolescents suicidants, et elle a conclu à une évolution plutôt favorable sur le plan psychosocial, puisque les trois quarts des participants s'estimaient heureux dans leur vie affective et plus de la moitié satisfaits dans leur vie professionnelle et 66,7% ne rapportent pas de trouble psychiatrique catégorisé. 24% des participants ont refait une ou plusieurs TS au cours des dix ans. Par ailleurs d'autres suicidants ont connu un profil évolutif moins favorable, des facteurs prédictifs de récidive suicidaire ont été signalés comme les difficultés et l'echec scolaire ainsi que la présence d'affections psychiatriques initiales [25]. En ce qui concerne le taux de répondeurs, notre étude a montré un taux de suivi très faible, 70% des suicidants sont perdus de vue. En effet dans notre société, consulter un psychiatre reste encore considéré comme tabou et stigmatisant pour la famille, ce qui limite beaucoup le suivi psychiatrique des suicidants.

## Conclusion

Les conduites suicidaires suscitent l'intérêt croissant de tous les professionnels de la santé, le risque principal est la prolongation d'une souffrance psychique qui s'exprime fréquemment par une récidive suicidaire et par un acte fatal. La prise en charge des suicidants demeure une priorité à travers le diagnostic et le traitement adapté des affections psychiatriques associées et avec un suivi régulier et un accompagnement après le geste suicidaire.
